# Pdx1 and Ngn3 Overexpression Enhances Pancreatic Differentiation of Mouse ES Cell-Derived Endoderm Population

**DOI:** 10.1371/journal.pone.0024058

**Published:** 2011-09-13

**Authors:** Atsushi Kubo, Robert Stull, Mitsuaki Takeuchi, Kristina Bonham, Valerie Gouon-Evans, Masayuki Sho, Masayuki Iwano, Yoshihiko Saito, Gordon Keller, Ralph Snodgrass

**Affiliations:** 1 First Department of Internal Medicine, Nara Medical University, Kashihara, Nara, Japan; 2 VistaGen Therapeutics, Inc., South San Francisco, California, United States of America; 3 The Department of Gene and Cell Medicine, Mount Sinai School of Medicine, New York, New York, United States of America; 4 First Department of Surgery, Nara Medical University, Kashihara, Nara, Japan; 5 McEwen Centre for Regenerative Medicine, Toronto, Canada; University of Southern California, United States of America

## Abstract

In order to define the molecular mechanisms regulating the specification and differentiation of pancreatic β-islet cells, we investigated the effect of upregulating Pdx1 and Ngn3 during the differentiation of the β-islet-like cells from murine embryonic stem (ES) cell-derived activin induced-endoderm. Induced overexpression of Pdx1 resulted in a significant upregulation of insulin (*Ins1* and *Ins2*), and other pancreas-related genes. To enhance the developmental progression from the pancreatic bud to the formation of the endocrine lineages, we induced the overexpression express of Ngn3 together with Pdx1. This combination dramatically increased the level and timing of maximal *Ins1* mRNA expression to approximately 100% of that found in the βTC6 insulinoma cell line. Insulin protein and C-peptide expression was confirmed by immunohistochemistry staining. These inductive effects were restricted to c-kit**^+^** endoderm enriched EB-derived populations suggesting that Pdx1/Ngn3 functions after the specification of pancreatic endoderm. Although insulin secretion was stimulated by various insulin secretagogues, these cells had only limited glucose response. Microarray analysis was used to evaluate the expression of a broad spectrum of pancreatic endocrine cell-related genes as well as genes associated with glucose responses. Taken together, these findings demonstrate the utility of manipulating *Pdx1* and *Ngn3* expression in a stage-specific manner as an important new strategy for the efficient generation of functionally immature insulin-producing β-islet cells from ES cells.

## Introduction

Islet transplantation has been shown to be useful in the treatment of patients with type 1 diabetes, even resulting in insulin independence [1], [2]. However, this therapeutic approach is limited by a shortage of transplantable islets. Consequently, other potential sources of β-islet cells are currently being sought. Two such alternatives are pancreatic duct cells and endocrine progenitor cells [3]–[5]. Another possible source of insulin-producing cells are pluripotent stem cells (ES or iPS), which are self-renewing and retain the potential to differentiate into all three germ layers [6], [7]. This makes pluripotent stem cells a very useful experimental model to study pancreatic development, and the pancreatic cells that develop from these stem cells are a potential source of islet cells for diabetes therapy.

Within the embryo, the pancreas derives from epithelium of the foregut endoderm, which forms dorsal and ventral buds on approximately day 9 of murine embryonic development [8], [9]. During pancreas and β-islet cell development, the transcription factor Pdx1 is expressed in the embryonic gut epithelium that will later give rise to the dorsal and ventral pancreas [10]–[12]. The fact that *Pdx1* is a master gene for early pancreatic development is demonstrated by the pancreatic agenesis occurring after bud formation in mice lacking functional *Pdx1* [13]. In addition, ectopic expression of Pdx1 induces pancreatic budding from the gut epithelium [14].

After pancreatic bud formation, another transcription factor, Neurogenin 3 (Ngn3), plays a critical role in the formation of pancreatic endocrine precursors. Notably, mice lacking *Ngn3* also lack the four pancreatic endocrine cells, which produce insulin (Ins), glucagon (Gcg), somatostatin (Sst) and pancreatic polypeptide (Ppy) [15]. Moreover, lineage tracking studies using the Cre-ER loxP system have shown that, after Pdx1 expression, Ngn3-positive cells give rise to all four endocrine cell types [16], suggesting that Ngn3 is expressed in the early endocrine progenitor cells that give rise to, and presumably contributes to the differentiation of, the four endocrine cell types. In addition, targeted disruption in mice has shown that various other transcriptional factors, including Pax4 [17], NeuroD [18], Nkx2.2 [19] and Nkx6.1 [20], are also critical for differentiation of endocrine progenitors into insulin producing β-islet cells. These factors must be expressed in a correct temporal order for appropriate lineage specification and differentiation of gut endoderm, pancreatic progenitors, endocrine progenitors and, finally, pancreatic β-islet cells.

We previously established a protocol for the activin-induced development of definitive endoderm during mouse ES cell differentiation [21], [22]. Similarly, D'Amour et al. reported that pancreatic hormone-expressing endocrine cells can be differentiated from human ES cell-derived endoderm through induction with activin [23], [24]. They further showed that the soluble growth factors that participate in pancreatic development during human embryonic development can mimic that process during human ES cell differentiation *in vitro* [23], [24].

In the present study, we evaluated the transcriptional regulation that is critical for induction of β-islet cell differentiation from mouse ES cell-derived endoderm. Previous study have demonstrated that biphasic induction of Pdx1 induce insulin producing cells in ES cell derived endoderm [25]. In this study, we show that temporally controlled expression of Pdx1 and Ngn3 induces pancreatic endocrine genes, various β-islet cell-related transcription factors, including Pax4, Pax6, Isl1 and Nkx2.2, and efficiently yields a high frequency of β-islet cells that express very high levels of insulin. Lastly, although these β-islet cells appropriately process and secrete insulin and C-peptide protein in response to various insulin secretagogues, they do not demonstrate adult levels of glucose-controlled insulin secretion.

## Results

### Pdx-1 induces insulin mRNA expression in activin-induced endoderm embryoid bodies (EBs)

It is well known that *Pdx1* is a master gene for early pancreatic development from the gut tube, and previous protocols resulted in very modest levels of *Pdx1*. Therefore, we engineered ES cells with a doxycycline (Dox)-inducible *Pdx1* (“tet-pdx1 ES cells”) expression vector to evaluate the effect of temporal control of *Pdx1* upregulation. For these studies we used Protocol #1 (“SP protocol”) as previously described [21]. *Pdx1* expression was induced in the cells by adding Dox (1 µg/ml) to the EB cultures between days 6 and 20.

**Figure 1:**
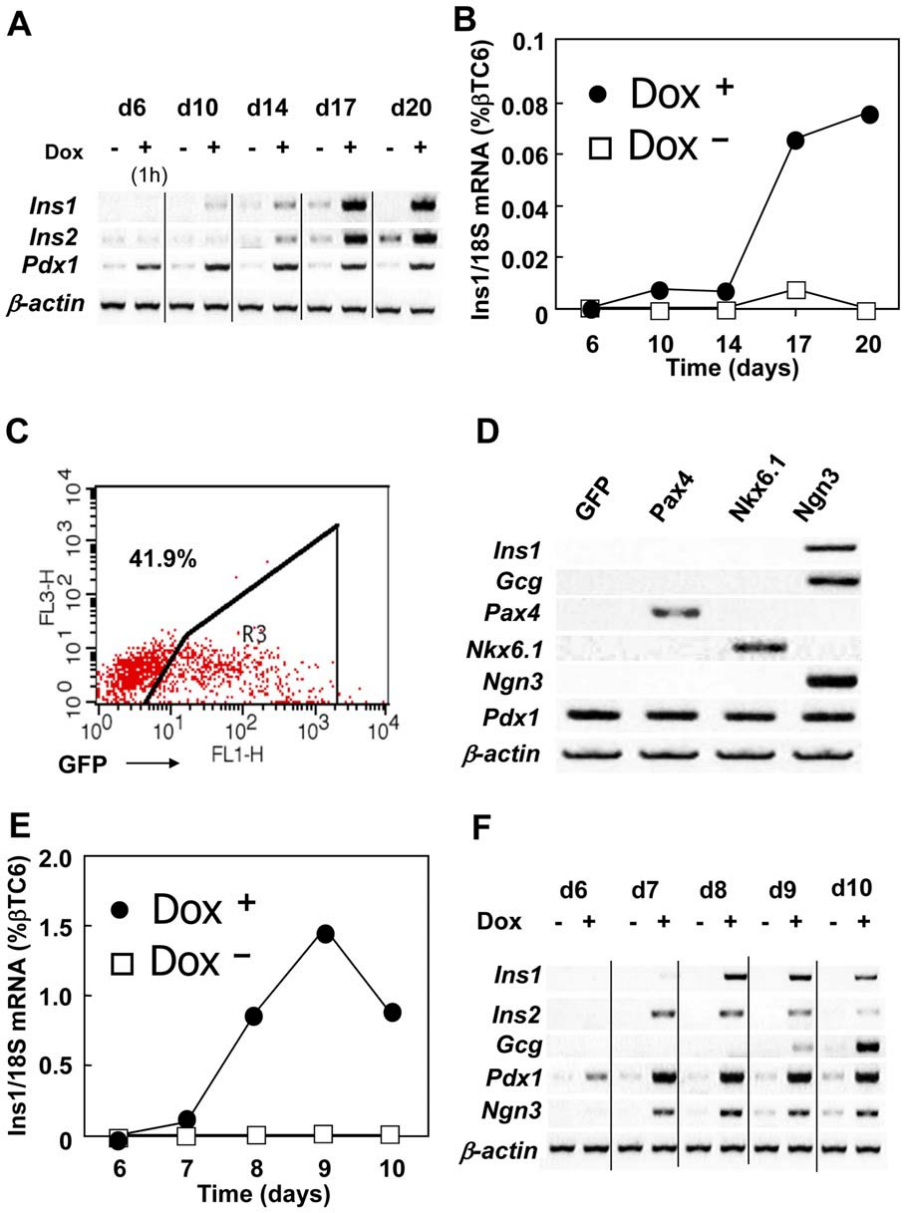
Pancreatic differentiation induced by Pdx1 and Ngn3. (A, B) Tet-pdx1 ES cells were cultured according to Protocol #1. Pdx1 expressions were induced with Dox (closed circle, Dox^+^) or without (open square, Dox^−^) for 6 to 20 days. A) Gene expression was analyzed by RT-PCR. B) *Ins1* mRNA levels were quantified by real time PCR and normalized to 18S mRNA levels. (C, D) EBs were differentiated for 6 days according to Protocol #1 and trypsinized to make single cell suspensions. The cells were electroporated with the pIRES2-EGFP vector and then reaggregated for 2–3 additional days with Dox. C) At day 8 (2 days with Dox), EGFP was evaluated by FACS. D) pIRES2 vectors, constructed to express GFP, Pax4, Nkx6.1 or Ngn3, were electroporated into day 6 EBs and allowed to reaggregate. At day 9, EBs were harvested and gene expression was analyzed by RT-PCR. (E, F) Tet-pdx1/ngn3 ES cells were cultured according to Protocol #1. Pdx1 and Ngn3 expression was induced with Dox (closed circle, Dox^+^) or without (open square, Dox^−^) at day 6 and harvested at the indicated time points. E) *Ins1* mRNA levels were quantified by real time PCR and normalized to levels in βTC6 as described in the Methods. F) Gene expression was analyzed by RT-PCR. Note, for the day 6 sample, the cells were exposed to Dox for only 1 hour before the samples were harvested.

Dox-induced *Pdx1* expression was confirmed by reverse transcription-polymerase chain reaction (RT-PCR) performed on days 6, 10, 14, 17 and 20 of differentiation (Fig. 1A). Overexpression of Pdx1 protein resulted in an upregulation of *Ins1* and *Ins2* mRNA expression seen as early as day 10 for *Ins1* and day 14 for *Ins2*, and they reached maximum levels at day 17 (Fig. 1A). *Ins1* is express only in pancreas, whereas *Ins2* is expressed in both pancreas and brain [26], [27]. Quantitative PCR analysis revealed that the normalized (see Methods) level of *Ins1* expression at day d20 represents 0.08% of the expression seen in the βTC6 insulinoma cell line (ATCC CRL-11506) (Fig. 1B). For comparison, normalized *Ins1* mRNA levels in mouse pancreatic islets are approximately 140% of the βTC6 levels (data not shown).

### Co-expression of *Ngn3* with *Pdx-1* increases insulin mRNA expression in activin-induced endoderm EBs

Because levels of *Ins1* mRNA are much lower in the ES differentiated cells than in βTC6 cells, and the fact that *Ngn3* levels in these cultures is very low (data not shown), we next evaluated the effects of *Ngn3* and other factors to improve β-islet cell differentiation and insulin expression. To do this, we transiently expressed target genes by cloning them into pIRES2-EGFP and electroporating the vectors into cells isolated from day 6 EBs under Protocol #1. Subsequent FACS analysis confirmed that this method produced about 42% GFP-positive cells 2 days after electroporation (Fig. 1C). Using this system, we transiently overexpressed *Pax4*, *Nkx6.1* and *Ngn3*, all of which are known to be important for β-islet cell specification, in cells with Dox-induced Pdx1 expression. RT-PCR or real time PCR on day 9 showed that all genes were expressed 3 days after electroporation and that only Ngn3 induced *Ins1* expression at significant levels (Fig. 1D). The transient upregulation of Ngn3 resulted in a significant increase above Pdx1 alone in the normalized expression of *Ins1* to approximately 0.07% of the βTC6 level, which was dependent on the Dox-induced upregulation of Pdx1 (data not shown). It is important to note that this transient Ngn3 expression, together with Pdx1, dramatically accelerated *Ins1* mRNA expression, and significantly higher levels of *Ins1* (data not shown).

To further characterize this effect, we generated Ainv cells capable of stable Dox-induced co-expression of *Pdx1* and *Ngn3* using a bi-cistronic *Pdx1*-IRES-*Ngn3* inducible expression construct. When Dox was added on day 6, normalized levels of *Ins1* mRNA increased to a maximum level by day 9, which was approximately 1.5% of the levels seen in βTC6 (Fig. 1E). Similarly, *Gcg* expression appeared on day 9 after upregulating both *Pdx1* and *Ngn3* (Fig. 1F). These data are consistent with the transient expression data presented in Figure 1D and suggest that stable co-expression of *Ngn3* and *Pdx1* increases *Ins1* mRNA levels about 20-fold higher than overexpression *Pdx1* alone, shortens the time to peak *Ins1* mRNA levels from 20 days to 9 days (compare Fig. 1B with 1E), and results in the differentiation of α-cell production of glucagon.

### Suspension culture in the presence of BMP4 enhances *Ins1* gene expression induced by *Pdx1* and *Ngn3*

**Figure 2:**
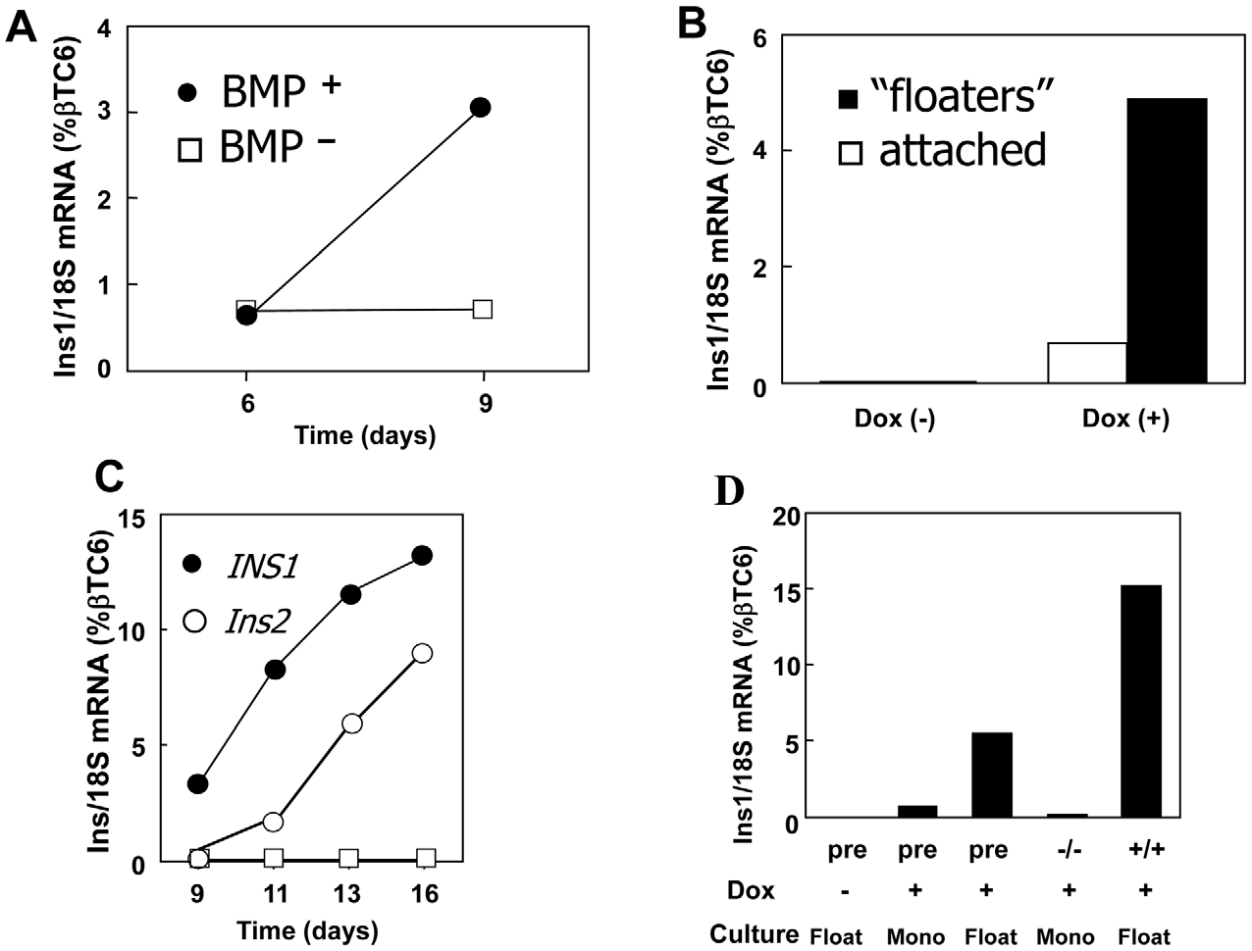
BMP4 and 3D non-adherent culture improves *Ins1* expression. Tet-pdx1/ngn3 ES cells were cultured according to Protocol #2. Pdx1 and Ngn3 were induced with or without Dox starting at day 4, and cells were harvested at the indicated time points. (A–D) insulin and 18S mRNA levels were quantified by real time PCR and normalized to levels seen in βTC6 as described in the Methods. A) Day 4 EBs were trypsinized and reaggregated with BMP4 (closed circles) or without (open squares). EBs were harvested at day 6 and 9. B) Day 4 EBs were trypsinized and reaggregated with BMP4. At day 6, EBs were replated on gelatin coated dishes and floating EBs were transferred to low-cluster dishes at day 7. Attached monolayer EBs (open bar) and floating EBs (closed bar) were harvested at day 9. (C) Floating EBs were cultured according to Protocol #2, with BMP4, and with Dox (circles) or without Dox (open squares). *Ins1* (black circles) or *Ins2* (white circles) mRNA levels were quantified by real time PCR and normalized to levels in βTC6 at the times indicated. (D) CXCR4/c-kit double negative (−/−) or double positive (+/+) cells were isolated by FACS from 4 days EBs, reaggregated for two days, and then replated on gelatin coated plates according to Protocol #2, with BMP4. Floating or attached EBs were harvested at day 9 from the following populations: pre-sorted cells (“Pre”); CXCR4/c-kit double negative (“−/−”); and CXCR4/c-kit double positive (“+/+”). These groups were cultured either as non-adherent EBs (“float”) or as attached monolayer cells (“mono”), as indicated.

Recently, Gouon-Evans et al. showed that BMP4 is required for hepatic differentiation of mouse ES cell-derived definitive endoderm [22]. The recent work of Wandzioch and Zaret also actually shows that in the mouse embryo, BMP is required at a later time point for pancreas specification compared to liver specification [28]. They show that an early activation with BMP4 would block pdx1 expression from endoderm (but induces alb expression), while is necessary few hours later to induce pdx1. These results suggested a dynamic role of BMP4 to induce liver and pancreatic differentiation [28]. In this study, we evaluated the effect of BMP4 on the differentiation of insulin producing cells from mouse ES cell-derived definitive endoderm. After two passages on gelatin, dissociated tet-pdx1/ngn3 ES cells were cultured for 2 days as EBs using Protocol #2 in serum-free differentiation medium, as previously described (“SFD protocol”) [22]. Activin A was then added from day 2 to day 4 to induce definitive endoderm, and on day 4, the effects of Dox and BMP4 were evaluated. Without Dox, no *Ins1* mRNA was detected on day 6 or 9 (data not shown). When Dox was added on day 4 to induce Pdx1 and Ngn3 expression, normalized *Ins1* mRNA levels reached 0.6% on day 6 and 3.1% on day 9 of differentiation of βTC6 levels (Fig. 2A). It was noticed that some of the day 6 EBs which were replated on gelatin coated 6 well plates attached on the plate and formed a monolayer, while others remained in suspension. The floating EBs were transferred to a low-cluster dish (12–24 well plate, Corning) on day 7. On day 9, all cells remained floating in these cultures and the *Ins1* mRNA levels were significantly higher in these cells than in the monolayer cells cultured on the gelatin coated dishes, reaching 4.9% of βTC6 levels (Fig. 2B). In a subsequent experiment, Dox was present continuously from day 4 and EBs were cultured with BMP4, bFGF and activin A from day 4 to day 6 and then transferred to low-cluster dishes (12- or 24-well) until day 16. Under these conditions expression of *Ins1* and *Ins2* mRNA continued to increase until day 16, reaching 13.2% and 8.2% of βTC6 levels, respectively (Fig. 2C). Thus supplementing the Protocol #2 with BMP4 and culturing in suspension increased *Ins1* mRNA levels about 10-fold, as compared to the Protocol #1.

### Insulin-expressing pancreatic cells are derived from a CXCR4/c-kit^+/+^ population

We previously showed that cells positive for both CXCR4 and c-kit give rise to definitive endoderm and hepatocytes [22]. Here we tested whether this same population also gives rise to pancreatic cells. EBs were cultured using Protocol #2 for 4 days, after which they were trypsinized to obtain suspensions of single cells, and immunostaining was then used to detect CXCR4 and c-kit. The stained cells were sorted and the CXCR4 and c-kit double positive and double negative populations were reaggregated for 2 days using Protocol #2. When reaggregated EBs were replated on gelatin-coated dishes on day 6 using Protocol #2, most double negative CXCR4/c-kit^(/)^ cells attached to the dishes, whereas most double positive CXCR4/c-kit^(**+**/**+**)^ endodermal cells did not attach but remained in suspension as EBs. On day 9, no *Ins1* mRNA was detected in the adherent monolayer of CXCR4/c-kit^(/)^ cells, whereas levels of *Ins1* mRNA in EBs from CXCR4/c-kit^(**+**/**+**)^ cells were more than 2-fold higher than in pre-sorted EBs (Fig. 2D). This suggests that, like ES-derived hepatocyte-like cells, ES-derived pancreas-like cells are derived from a CXCR4/c-kit^(**+**/**+**)^ definitive endoderm population, which differentiates more efficiently in a 3-dimensional non-adherent structure as opposed to a 2D monolayer. Interestingly, these isolated endodermal cells produced small EBs that did not grow well. We could not continue to culture the 3-deminsional non-adherent structures beyond day 9. They did not grow well and tended to disrupt. We suspect that there may be a requirement for a non-endodermal cell for maintenance and growth of the pancreas cells under these conditions.

### Pancreas-related genes induced by Pdx1 and Ngn3 under Protocol #2 with BMP4

**Figure 3:**
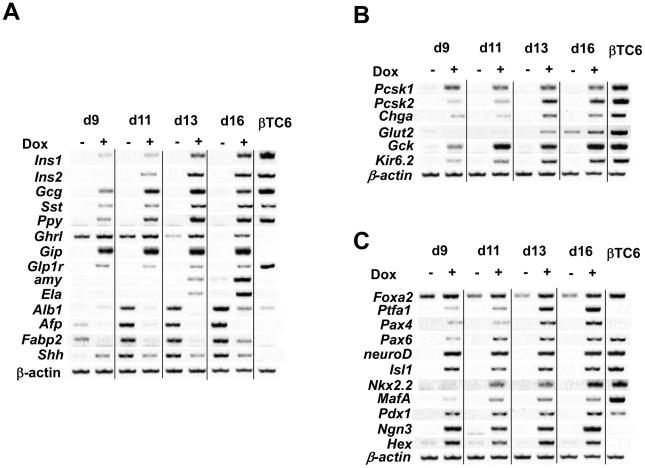
Time course of pancreas-related gene expression. Tet-pdx1/ngn3 ES cells were cultured according to Protocol #2, with BMP4. *Pdx1* and *Ngn3* were induced with Dox (Dox^+^) or without (Dox^−^) at day 4, and gene expression was analyzed from day 9–16 by RT-PCR and compared to βTC6 mRNA. (A) Secretory proteins and liver/intestine related-genes. (B) Insulin processing genes and glucose sensing genes. (C) Pancreas related-transcriptional factors.

RT-PCR was used to evaluate the expression of *Ins1* and a variety of pancreas-related genes from cells cultured in suspension with BMP4 under Protocol #2. These studies revealed that overexpression of Pdx1 and Ngn3 induced the following genes: 1) secretory pancreatic endocrine genes (*Ins1*, *Ins2*, *Gcg*, *Sst*, *Ppy* and *Ghrl*), incretin hormone related-genes (*Gip* and *Glp1r*), and exocrine genes (*Amy* and *Ela*) (Fig. 3A); 2) genes related to insulin secretion and insulin processing (*Pcsk1*, *Pcsk2* and *Chga*), genes related to glucose sensing (*Glut2* and *Gck*), and genes related to potassium ion channels (*Kir6.2*) (Fig. 3B); and 3) pancreas-related transcription factor genes (*Ptfa1*, *Pax4*, *Pax6*, *neuroD*, *Isl1*, *Nkx2.2*, *MafA* and *Hex)* (Fig. 3C). It is interesting to note that the liver- and intestine-related genes *Alb*, *Afp* and *Fabp2* were suppressed by Dox induction, as was *Shh*, an inhibitor of pancreatic endoderm (Fig. 3A).

### Microarray analysis of genes downstream of Pdx1 and Ngn3

For a more in-depth analysis of the impact of Pdx1 and Ngn3 expression on lineage development, we carried out a microarray analysis to identify genes activated downstream of Pdx1 and Ngn3. For these studies, gene expression in Dox-stimulated tet-pdx1/ngn3 Ainv cells cultured under Protocol #2, with BMP4 and floating non-adherent conditions for 13 days, were evaluated by microarray analysis and compared to gene expression of E15.5 embryonic pancreas, adult islet cells, and βTC6 cells. All primary microarray data are available at http://www.ncbi.nlm.nih.gov/geo/ under accession numbers GSM586113-122 and GSM312098-127.

With this analysis we found that other pancreas-related genes are upregulated by Pdx1 and Ngn3 (supplemental Table S3). These genes were categorized based on gene ontology analysis as follows: 1) “Extracellular” secretory proteins including: A) pancreatic endocrine and exocrine genes (*Ins1*, *Sst*, *Gcg*, *Ppy*, *Ghrl* and *Cpa*); B) genes related to insulin secretion (*Scg*, *Chga*, *Pcsk*); and C) enteroendocrine genes (*Gip*, *Cck*, *Pyy*, *Sct* and *Ghrl*); 2) “Nuclear” transcription factors, including β-islet cell-related transcription factors (*Pax6*, *Insm1*, *Neurod1*, *Nkx2.2*, *Isl1*, *Hhex*, *Nkx6.1* and *Pax4*) and α-cell-related transcription factors (*Arx*, *Irx2*); and 3) “Miscellaneous”, which includes genes whose direct functional role in β-islet differentiation is not clear, i.e. cytoskeletal/membrane and cytoplasmic/signaling genes. The upregulation of some of the Miscellaneous genes, e.g. (*Dcx*, *Stmn2* and *Tubb3*), is consistent with an earlier study evaluating novel Ngn3-related genes in mouse ES cells [29]. To determine the time course of the expression of these genes under Protocol #2 culture conditions, microarray analyses were performed on days 4, 6 and 13. Selected pancreas-related genes (*Ins1, Gip, neurod1, Sst, Scg3, Insm1, Gcg, Pax6, and Arx*) indicate that there is a significant upregulation of these genes after day 6, suggesting differentiation from endoderm to a pancreatic fate during this time period (Supplemental Fig. S1).

### Depletion of N2 and RA increases *Ins1* mRNA levels

**Figure 4:**
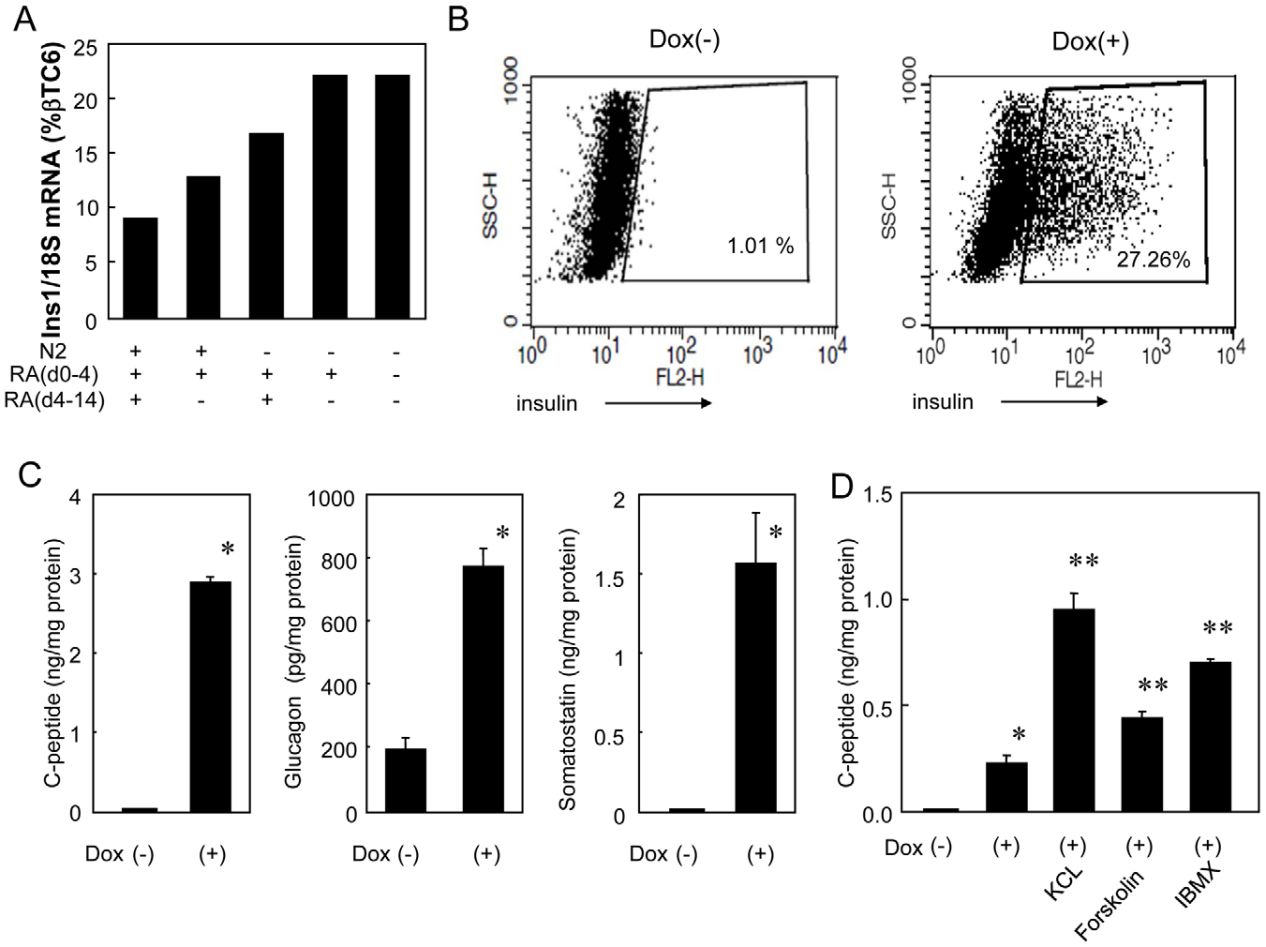
Deletion of N2 and RA supplements increases *Ins1* expression. Tet-pdx1/ngn3 ES cells were cultured with Dox (Dox^+^) or without (Dox^−^) according to Protocol #3, including B27 supplement formulated with or without RA as indicated. (A) N2 supplement was added to the indicated groups from the beginning through day 14. B27(+RA), indicated by “+”, or B27(−RA), indicated by “−”, was included as a supplement from either day 0–4 or from day 4–14 as indicated. *Ins1* mRNA levels at day 14 were quantified by real time PCR and normalized to βTC6 levels. (B) Tet-pdx1/ngn3 ES cells were cultured with Dox (Dox^+^) or without (Dox^−^) according to Protocol #3 for 18 days. EBs were trypsinized to make single cell suspensions and cytoplasmic insulin was detected by immunostaining and analyzed by FACS. (C) EBs were cultured according to Protocol #3 for 18 days with or without Dox, after which the media was replaced with fresh medium for 24 hours and supernatants were harvested. C-peptide, glucagon and somatostatin were measured by either RIA (C-peptide) or EIA (glucagon, somatostatin) as described in Materials & Methods. (D) EBs cultured according to Protocol #3 for 19 days were resuspended in HKRB buffer and stimulated with KCl (30 mM), Forskolin (10 M) or IBMX (0.5 mM) for 1 hour. Supernatants were harvested and C-peptide was measured by RIA. For the Dox^−^ samples in panels C & D, C-peptide and somatostatin values were not detectable above background. The data presented are mean of three independent experiments; the error bars represent the SEM. *p<0.05 as compared with Dox (−), **p<0.05 as compared with Dox(+).

Protocol #2 used in this study was originally established for hepatocyte differentiation from mouse ES cells [22]. To optimize **β**-islet cell differentiation we tested whether N2 or RA, supplements that we used for differentiating hepatocytes, were useful for pancreatic EB differentiation and *Ins1* expression. We explored this with Protocol #3, which is identical to Protocol #2 except that N2 and B27 (−RA, i.e.-without RA), were not used at any time during the culture, and after day 6 the cells were grown in suspension (see Supplemental Table S1). We found that the highest *Ins1* expression was obtained when both N2 and RA were removed from the protocol, which resulted in an increased *Ins1* mRNA level to 23% of that in **β**TC6 cells (Fig. 4A). FACS analyses indicated that approximately 27% of Dox-stimulated EBs cultured under Protocol #3 for 18 days showed cytoplasmic insulin staining (Fig. 4B). This insulin expression was dependent on the expression of Pdx1 and Ngn3 since only 1% of EBs cultured in the absence of Dox were positive for insulin (Fig. 4B).

### C-peptide is secreted from EBs induced by Pdx-1 and Ngn3

To evaluate pancreatic hormone secretion, pancreatic EBs were cultured for 18 days under Protocol #3, after which the medium was replaced with fresh Protocol #3 medium (IMDM,F12,BSA,B27(−RA)) for 24 hours. We then assayed the supernatant for C-peptide, glucagon and somatostatin using RIAs or EIAs. In the absence of Dox stimulation, no C-peptide, somatostatin or glucagon was detected. By contrast, all three were detected in supernatants conditioned by EBs stimulated with Dox (Fig. 4C). We also assessed the ability of various secretagogues to stimulate secretion of C-peptide (Fig. 4D). We found that stimulation with 30 mM KCl for 1 hour increased C-peptide secretion about 5-fold. Similarly, forskolin and IBMX, which increase intracellular cAMP levels, also increased C-peptide secretion approximately 2-fold and 3-fold, respectively. On the other hand, we detected no response to glucose or to the K_ATP_ channel inhibitors glibenclamide and tolbutamide (data not shown). This suggests that whereas pancreatic EBs induced by Pdx1 and Ngn3 are capable of responding to stimuli that evoke secretion directly (i.e., membrane depolarization or an increase in intracellular cAMP), they lack the machinery to respond to glucose or a K_ATP_ channel inhibitor.

### Immunohistochemical analysis of pancreas-related proteins

**Figure 5:**
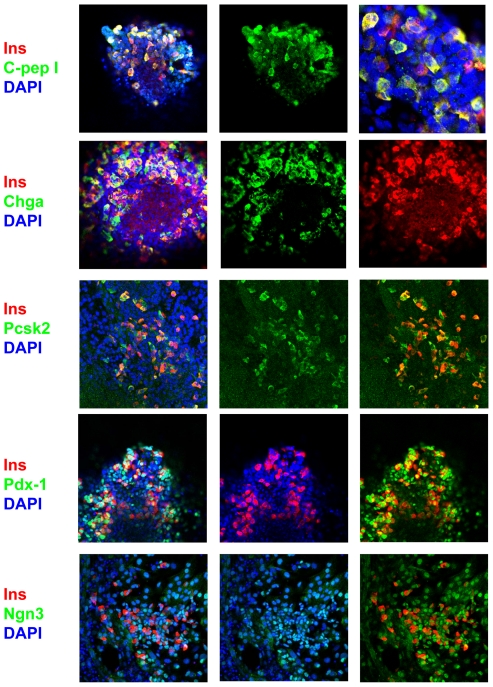
Endocrine protein expression in pancreatic EBs induced by Pdx1 and Ngn3. Tet-pdx1/ngn3 ES cells were cultured according to Protocol #3. At day 16, EBs were replated on glass bottom dishes coated with Matrigel. Replated EBs were stained with antibodies for the indicated markers of endocrine cells. Insulin was visualized with a Cy3-conjugated 2^nd^–stage fluorescent antibody (red) and other markers were visualized with a FITC-conjugated 2^nd^–stage fluorescent antibody (green). Nuclei were stained with DAPI (blue). Magnification of right panel for C-peptide and insulin was with a 1000× objective. Magnification for the other panels was with a 400× objective.

We next carried out an immunohistochemical analysis to evaluate protein expression in EBs in which Pdx1 and Ngn3 were induced. Tet-pdx1/ngn3 ES cells were cultured for 16 days under Protocol #3, with or without Dox, and then replated on glass bottom dishes coated with Matrigel. On day 18, the EBs were immunostained and examined using confocal microscopy. We found that insulin, C-peptide, Chga and Pcsk2 proteins are all expressed in Dox-stimulated EBs (Fig. 5), whereas no staining is detected in EBs not stimulated with Dox (data not shown). Importantly, most insulin-positive cells co-expressed C-peptide. As positive controls, we also stained for Pdx1 and Ngn3 after Dox stimulation (Fig. 5). Our results suggest that the temporal upregulation of Pdx1 and Ngn3 induces endocrine pancreas cells that produce **β**cell-related proteins. The fact that detecting insulin protein and C-peptide was dependent on Dox-regulation indicates that these results are detecting endogenously produced insulin and are not subject to previous published insulin artifacts [30].

### Removal of B27 from DMEM enhances pancreatic differentiation

**Figure 6:**
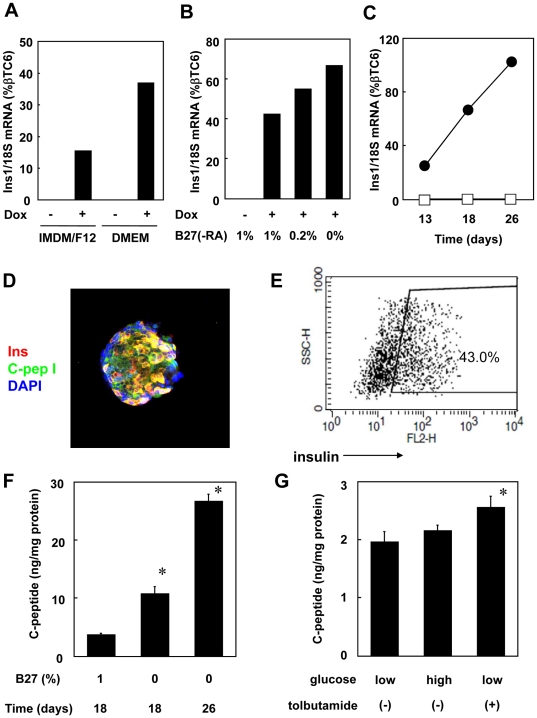
DMEM media improves pancreatic differentiation of mouse ES cells. Tet-pdx1/ngn3 ES cells were cultured under Protocol #3 for 6 days, with Dox-induction of Pdx1 and Ngn3 expression starting at day 4. A) From day 6 through day 18 EBs were cultured according to Protocol #3 (“IMDM/F12”) or in medium in which the IMDM/F12 was replaced with DMEM (“DMEM”) as indicated (Protocol #4), and *Ins1* mRNA was quantitated at day 18. B) EBs were cultured according to Protocol #4 for the first 6 days and with Dox-induction of Pdx1 and Ngn3 expression starting at day 4. From day 6 through day 18 various concentrations of B27(−RA) were added as indicated. *Ins1* mRNA was quantitated at day 18. C) EBs were cultured according to Protocol #4 for 6 days, with Dox-induction of Pdx1 and Ngn3 expression starting at day 4. From day 6 through day 26 the medium was only DMEM with BSA without B27(−RA). *Ins1* mRNA was quantitated from day 13–26. (D–G) Tet-pdx1/ngn3 ES cells were cultured according to Protocol #4 for 24 days with Dox induction starting at day 4. D) EBs were replated at day 24 and cultured in DMEM (BSA). At day 26, Insulin was visualized with a Cy3-conjugated 2^nd^ antibody (red) and C-peptide was visualized with a FITC-conjugated 2nd antibody (green). Nuclei were stained with DAPI (blue). Magnification was with a 400× objective. E) EBs, prepared as in panel D, were trypsinized to make single cell suspensions and cytoplasmic insulin was stained and analyzed by FACS. F) EBs were cultured under Protocol #4 for 6 days, with Dox-induction of Pdx1 and Ngn3 expression starting at day 4. From day 6 through day 18 only one group was supplemented with B27(−RA) (1%) as indicated. On either day 18 or day 26 the media was replaced with fresh DMEM with BSA. Supernatants were harvested after 24 hours, and C-peptide was measured by RIA. The data presented are mean of three independent experiments; the error bars represent the SEM. p<0.05 as compared with 1% B27 at day 18. G) EBs were prepared as described in panel F. On day 26 the media was replaced with HKRB buffer containing either low glucose media (2 mM) or high glucose media (20 mM) with or without tolbutamide. Supernatants were harvested after 1 hour and C-peptide was measured by RIA. The data presented are mean of three independent experiments; the error bars represent the SEM. p<0.05 as compared with low glucose without tolbutamide.

After culturing tet-pdx1/ngn3 ES cells under Protocol #3 for 6 days, we cultured the cells for the next 12 days according to Protocol #3 or a modified Protocol #3 in which the IMDM/F12 was replaced with DMEM (Protocol #4). We found that levels of *Ins1* mRNA were higher in the group that contained DMEM instead of IMDM (Fig. 6A). Moreover, using different concentrations of B27(−RA) according to Protocol #4 revealed that eliminating B27(−RA) after day6 resulted in an increased *Ins1* expression that reached 66% of βTC6 levels on day 18 (Fig. 6B). *Ins1* mRNA ultimately reached 100% of the level in βTC6 cells by day 26 (Fig. 6C). Immunostaining showed that there was a higher fraction of cells positive for both insulin and C-peptide when cells were cultured according to Protocol #4, without B27(−RA) after day6, compared to Protocol #3 (compare Fig. 6D with Fig. 5). Using FACS analyses, we also confirmed that 43% of cells from Dox-stimulated EBs cultured under Protocol #4, without B27, showed cytoplasmic insulin (Fig. 6E), whereas only 1.0% of EBs cultured in the absence of Dox were insulin-positive (data not shown). Under these conditions the 24 hour secretion of C-peptide increased approximately 4-fold and 10-fold on days 18 and 26, respectively, as compared to Protocol #3 (compare Fig. 6F with Fig. 4C). Although these conditions resulted in very high *Ins* transcription levels and frequency of insulin positive cells, this increased level of C-peptide secretion was still less than 1% of the level of C-peptide secreted from βTC6 cells, which was 3766±82 ng/mg protein/24 hour (data not shown). Under these conditions, tolbutamide significantly increased C-peptide secretion measured at one hour of stimulation, whereas high glucose did not (Fig. 6G).

## Discussion

Efficient endodermal differentiation is a critical first step in pancreatic development, since the pancreas is an endoderm-derived tissue. We previously established two types of differentiation protocols for generating definitive endoderm from ES cells [21], [22]. We show here the utility of temporally upregulating the expression of Pdx1 and Ngn3, which are known to be important for pancreatic development, can induce at high frequency the differentiation of endodermal cells into β-islet cells that transcribe insulin at high levels, and that these cells secrete C-peptide, respond to insulin secretagogues, but do not respond to glucose.

When Pdx1 is upregulated after the induction of endoderm differentiation, the transcription of both *Ins1* and *Ins2* increased, and when both and *Pdx1* and Ngn3 were co-expressed, *Ins1* and *Ins2* transcription was upregulated to a significantly greater extent, reaching maximal levels in half the time. These results are consistent with a large body of evidence of the importance of Pdx1 and Ngn3 expression for the normal development of β-islet cell function and insulin expression. Pdx1 is first expressed in the pancreatic bud of the gut tube [10], [12]. All pancreatic cell types are derived from Pdx1-positive progenitors [16], and Pdx1 is required for *Ins* transcription [10], [31], [32]. Previous reports have shown that Ngn3 is expressed in the embryonic pancreas at E10.5, and mice lacking *Ngn3* fail to generate pancreatic endocrine cells [15]. In addition, cell tracking experiments showed that all β-islet cells are derived from precursors that express Pdx1 before they express Ngn3 [16], [33], and that the four types of islet endocrine cells are derived from Ngn3-positive precursors [16]. These results suggest that Pdx1 is involved in the induction of endoderm to a pancreatic fate, after which Ngn3 induces the specification and differentiation of pancreatic endocrine cells from pancreatic primordium, and Pdx1 continues to be required for insulin expression and normal β-islet cell function. Moreover, expression of Pdx1 and Ngn3 was recently reported to be key events in β-islet cell regeneration from pancreatic duct cells in vivo [34]. These results, together with the results presented here, suggest that the same machinery may be important for fetal islet development, adult islet regeneration, and ES cell-derived pancreatic differentiation.

To optimize the conditions that promote ES-derived pancreatic differentiation, we evaluated Protocol #2. Gouon-Evans et al. originally developed this protocol (“SFD”) for mouse ES-derived hepatocyte differentiation and showed that BMP4 is required for hepatic differentiation under this protocol [22]. It is important to note that all of the protocols we have studied require activin to initiate high efficiency generation of endoderm. Here we demonstrate that exogenous BMP4 improves mouse ES-derived pancreatic differentiation and increases *Ins1* mRNA levels. BMP4 also reportedly promotes the proliferation and development of epithelial islet-like structures and pancreatic progenitor cells *in vitro* [35], [36]. The recent work of Wandzioch and Zaret also actually shows that in the mouse embryo, BMP is required at a later time point for pancreas specification compared to liver specification [28]. In the present study, we found that changes to the Protocol #2 culture medium, namely adding BMP4, using DMEM instead of IMDM/F12, eliminating the N2 and B27 supplements, culturing the cells in 3D non-adherent structures, i.e. Protocol #4, dramatically increased *Ins1* mRNA levels up to 100% of the normalized values seen in βTC6 insulinoma cell line. Under these conditions, although the EB size was smaller than observed with Protocol #2, the relative number of insulin positive cells increased. This suggests that Protocol #4 provides a relative enrichment for insulin-positive cells. EBs induced toward pancreatic differentiation by the above protocols express a variety of pancreas-related proteins detected immunohistochemically, including Chga, Pcsk2, plus fully processed insulin and C-peptide. In addition, insulin was processed and C-peptide was secreted into the supernatant. This suggests that Pdx1 and Ngn3 induce efficient differentiation of functional islet-like cells from ES cell-derived endoderm and that the cells are capable of producing and correctly processing insulin.

This *in vitro* differentiation process mimics embryonic pancreatic endocrine development, which can be useful for evaluating the gene expression pattern of pancreatic endocrine cell differentiation and β-islet cell biology. To evaluate this, we performed microarray analyses of Pdx1- and Ngn3-induced EB differentiation. Through these analyses we found that pancreas-related genes encoding secretory proteins (e.g., *Ins* and *Sst*) and transcription factors (e.g., *Pax6 and neuroD*) were induced by Pdx1 and Ngn3 during EB differentiation. Conversely, genes characteristic of hepatocyte and intestine were suppressed by Pdx1 and Ngn3. This accurately reflects the known functional tissue specificity of these genes. In addition, other genes recently reported to play a role in pancreas development were also shown to be upregulated in Pdx1- and Ngn3-induced EBs. For example, cocaine- and amphetamine-regulated transcript (*Cart*) is upregulated in our studies and was recently reported to be expressed in pancreatic β-islet cells and to enhance insulin secretion from an insulinoma cell line and from isolated rat islet cells [37], [38]. Resp18 is increased 87-fold by Pdx1/Ngn3 induction in our studies and was also recently reported to be expressed in pancreatic β-islet cells and markedly stimulated following exposure to high glucose [39]. We also found that genes expressed in enteroendocrine cells (e.g., *Gip*, *Cck*, *Pyy* and *Ghrl*) were upregulated, suggesting that these genes important for both enteroendocrine and pancreatic hormones are dependent on the expression Pdx1 and/or Ngn3.

Consistent with normal β-islet cell biology, direct depolarization of the ES-derived cells using KCl, or IBMX- or forskolin-induced intracellular cAMP increase, stimulated C-peptide release from the ES-derived pancreatic cells during a 1-hour secretion assay. These combined results indicate that ES-derived pancreatic cells produce and correctly process insulin and that C-peptide secretion is regulated by various insulin secretagogues. Despite the fact that *Ins1* mRNA levels were comparable to those found in βTC6 cells in protocol #4 differentiated cells, C-peptide secretion was not glucose responsive and was only 0.75% of the normalized levels observed in βTC6 cells. Apparently, the current protocol does not induce at high efficiency the final maturation steps that result in adult-level glucose responsiveness. By using various growth factors at each differentiation step, D'Amour et al. were able to differentiate insulin-expressing cells from human ES cells [24] that could respond to glucose in vivo and could be used to successfully treat diabetic mice, suggesting that they were functionally glucose responsive [40]. It is not clear from D'Amour's studies if the β-islet cells were glucose responsive *in vitro* prior to transplantation *in vivo*. It is possible that in vivo conditions are required for the final maturation steps leading to fully mature functional properties, which are not yet replicated in our culture systems. The identification of the potential biological factors and/or cellular interactions that are required for final β-islet cell maturation and full glucose responsiveness remains an elusive problem and as yet not replicated *in vitro*.

In conclusion, we have shown that mouse ES cells can be induced by activin to differentiate into definitive endoderm, followed by differentiation to pancreatic endocrine cells through subsequent stimulation of BMP4 and overexpression of Pdx1 and Ngn3. During this differentiation scheme, many gene expression cascades that mimic embryonic pancreatic development are expressed in an appropriate temporal order, suggesting this *in vitro* model may be useful as a screen for evaluating potential genes, or drug candidates, that induce or increase pancreatic endocrine cell differentiation. In addition, it is clear that further work is necessary to define the requirement for efficient and reproducible induction of full β-islet cell maturation and glucose responsiveness *in vitro*. These studies also highlight some of the limitations of current differentiation protocols as well as offer improved protocols that dramatically improve the frequency and level of insulin expression. These studies further support the view that ES cells, and iPS by extension, have the potential to be a renewable resource for producing β-islet cells for cell-based diabetes therapy.

## Methods

### Growth and Differentiation of ES cells

We used Ainv 18 mouse ES cells (a kind gift from Drs. Michael Kyba and George Daley; ATCC SCRC-1029) to assess gene function during ES cell differentiation to pancreatic lineages [41]. These cells were engineered to allow “cre-lox” targeted insertion of “tet-on” expression cassettes into the hprt locus. This system enables the efficient temporal upregulation of target genes by the addition of Dox (Sigma, St. Louis). For some experiments, the bi-cistronic expression of two target genes was achieved through the use of an intervening IRES sequence [42]. *Pdx1* or pdx1-IRES-ngn3 pLOX expression vectors were electroporated, together with a cre expression construct [43], into Ainv 18 ES cells according to published procedures (Amaxa, Lonza, AG). This yielded “tet-pdx1” or “tet-pdx1/ngn3” ES cells, which could be induced by Dox to express *Pdx1* or both *Pdx1* and *Ngn3*, respectively. ES cells were maintained on irradiated mouse embryo fibroblast feeder cells as previously described [21]. To generate EBs, ES cells were passaged two times on gelatin, dissociated into a suspension of single cells using trypsin (0.25%), and then cultured at various concentrations in 60 mm bacterial-grade petri dishes (Valmark) in differentiation media, as described below. Cultures were maintained in a humidified chamber under a 5% CO_2_-air mixture at 37°C.

Four protocols were evaluated for differentiation of pancreas-like cells (see details in Supplemental Table S1). The first protocol utilized activin-mediated induction of endoderm via a two-step endoderm Protocol #1 (“SP protocol”) as previously described [21]. Dissociated undifferentiated ES cells (4×10^3^ cells/ml) were cultured for 2 days in suspension in Stem Pro 34 medium (Gibco) supplemented with 2 mM glutamine, 0.5 mM ascorbic acid, 4.5×10^−4^ M monothioglycerol (MTG) and c-kit ligand (1% conditioned medium from CHO cells). EBs were harvested after 48 hours of differentiation, allowed to settle in a 50 ml tube, transferred to new petri dishes (Valmark) and cultured for 4 days in IMDM supplemented with 15% Knockout serum replacement (SR) (Gibco), 2 mM glutamine, 0.5 mM ascorbic acid, 4.5×10^−4^ M MTG and human activin A (100 ng/ml) (R&D Systems). To induce test genes, starting on day 6 after EB formation, Dox (1 µg/ml) was included in IMDM supplemented with 15% SR, 2 mM glutamine, 0.5 mM ascorbic acid, and 4.5×10^−4^ M MTG. After differentiation for 10 days from the start of differentiation, the EBs were transferred to Matrigel-coated 6-well dishes (Falcon) in IMDM supplemented with 15% fetal calf serum (FCS; JRH) and 2 mM glutamine, with or without Dox (1 µg/ml). Cells from these replated cultures were harvested for RNA isolation at the indicated times from the start of initial differentiation.

Protocol #2 used serum-free differentiation medium, as previously described (“SFD protocol”) [22], that contains 75% IMDM and 25% Ham's F12 medium (Gibco) supplemented with 0.5% N2 and 1% B27 (with vitamin A/retinoic acid, “RA”) (Gibco), 1% penicillin/streptomycin, 0.05% bovine serum albumin, 2 mM glutamine, 0.5 mM ascorbic acid and 4.5×10^−4^ M MTG. ES cells (2–4×10^4^ cells/ml) were cultured in this medium in 60 mm low attachment petri dishes. On day 2 of differentiation, EBs were dissociated with trypsin/EDTA and replated at a density of 2–6×10^4^ cells/ml in new 60 mm petri dishes using Protocol #2 medium supplemented with activin A (50 ng/ml). On day 4, EBs were dissociated with trypsin/EDTA and then reaggregated at high density (5×10^5^ cells/ml) in 24-well low-cluster dishes (Nunc) in Protocol #2 medium supplemented with BMP4 (50 ng/ml) (R&D Systems), bFGF (10 ng/ml) (R&D Systems) and activin A (50 ng/ml), with or without Dox (1 µg/ml). On day 6, EBs were replated on gelatin coated 6-well dishes (Falcon) for monolayer culture or in 12-well low-cluster dishes (Nunc) to obtain non-adherent floating EBs in Protocol #2 medium, with N2 and/or B27(+RA), without either ascorbic acid or MTG, and with or without Dox (1 µg/ml). Protocol #3 was identical to Protocol #2 except B27 did not contain RA, media was not supplemented with N2, and after day 6 the cells were cultured in low-cluster dishes to obtain non-adherent floating EBs. Protocol #4 was identical to Protocol #3 except DMEM was used instead of IMDM/F12 media.

### PCR Gene expression analysis

For RT-PCR, total RNA was extracted using RNeasy mini-kits and treated with RNase-free DNase (Qiagen). One µg of total RNA was reverse-transcribed to cDNA using a Superscript RT kit (Invitrogen) with random hexamers. PCR was carried out using Taq polymerase (Takara Bio) in PCR buffer containing 2.5 mM MgCl_2_ and 0.2 µM dNTPs. The amplification protocol was 1 cycle at 94°C for 5 min followed by 25–40 cycles of 94°C for 1 min (denaturation), 60°C for 30 sec (annealing) and 72°C for 1 min (elongation), with a final elongation at 72°C for 7 min. The oligonucleotide primers used for PCR are listed in supplemental Table S2.

For quantitative real-time PCR, commercially available assay mixes (Applied Biosystems) for *Ins1* (Mm01259683_g1), *Ins2* (Mm00731595_gH) and *18S* (Hs99999901_s1) were used to quantify mRNA levels, and PCR was performed using a Prism 7700 Sequence Detector (Applied Biosystems). *Ins1* and *Ins2* mRNA levels were normalized to *18S* mRNA levels in the same samples, expressed as *Ins*/*18S* ratios, and plotted as percentages of the *Ins*/*18S* ratios observed in βTC6 cells grown in 15%FCS DMEM and harvested in log phase. All experiments repeated at least two times.

### Transient gene upregulation mediated by vector electroporation

*Pax4*, *Nkx6.1* and *Ngn3* coding regions were cloned downstream of the CMV promoter in the pIRES-EGFP vector (Clontech). Tet-pdx1 or tet-pdx1/ngn3 ES cells were cultured under Protocol #1. On day 6, EBs were dissociated with trypsin/EDTA (0.25%), after which the resultant cells (1–3×10^6^ cells) were suspended in mouse ES cell nucleofection solution (Amaxa). Gene expression plasmids (5 µg) were electroporated into cells using an Amaxa Nucleofector device (ES solution, program O-17). The cells were then washed and reaggregated in 24-well low-cluster dishes (Costar) in IMDM supplemented with 15% SR (Gibco), 2 mM glutamine, 0.5 mM ascorbic acid and 4.5×10^−4^ M MTG containing Dox (1 µg/ml). EBs were harvested on day 8 for FACS or on day 9 for RNA isolation.

### Microarray analysis

For microarray analysis, total RNA was extracted using RNeasy mini kits (Qiagen), after which 10-µg samples of fragmented target total RNA was used for hybridization of CodeLink Mouse Whole Genome Arrays (GE Life Sciences), which contained 34,967 probes. Once the microarrays were hybridized and washed, biotin-containing transcripts were directly detected using a Streptavidin-Alexa647 conjugate as previously described [44]. GeneSpring 7 (Silicon Genetics, Inc., Redwood City, CA) was then used to evaluate the data obtained using CodeLink™ Expression Scanning Software. Because some CodeLink probes are improperly annotated as to their intended target, refinement of gene-to-probe associations was accomplished by analysis using VistaGen's Valid Probe Set tools, which results from a mapping of the genomic coordinates of probes with that of the exons of genes. All genomic coordinates of the mouse genome were determined using BLAST. Invalid probes, such as those that target multiple genes, non-coding strand, or intergenic regions on the genome, were removed from analyses.

### FACS analysis and cell sorting

We used our previously published methods to isolate definitive endoderm populations [22], [45]. Day 4 EBs were trypsinized for single cell suspension, then stained with a PE-conjugated anti-c-kit antibody (BD Pharmingen, 553355, 1/150) and a biotinylated rat anti-mouse CXCR4 antibody (BD Pharmingen, 551968, 1/200), and visualized using streptoavidin PE-Cy5 (BD Pharmingen). Cytoplasmic insulin was detected in cells from day 18 EBs dissociated with 0.25% trypsin/EDTA and 0.05% collagenase, stained with a guinea pig anti-insulin antibody (Dako, A0564 1/100), and visualized using a PE-conjugated anti-guinea pig IgG secondary antibody (Jackson Immunoresearch) with the Cytofix/Cytoperm kit (Becton Dickenson) according to manufacturer's recommendations. The stained cells were also analyzed using a FACScan (Becton Dickinson, San Jose, CA) or sorted on a FACS Aria cell sorter (Becton Dickinson). All experiments repeated at least two times.

### Immunostaining

For immunostaining, day 16 EBs were plated on glass bottom dishes (Matek) coated with Matrigel. Two days later, the day 18 EBs were fixed in 4% paraformaldehyde for 20 min, washed two times in PBS, permeabilized in PBS containing 0.2% triton-×100, washed in PBS containing 10% FCS and 0.2% Tween 20, and then blocked for 10 min in PBS containing 10% horse serum. The cells were then incubated for 1 hour with a primary antibody specific for either insulin (Dako, A0564, 1/150), C-peptide (Yanaihara, Y222, 1/100), Pdx1 (Transgenic, KR059, 1/100), Ngn3 (Santa Cruz sc-25655, 1/50), Pcsk2 (Chemicon, AB1262, 1/100) or Chga (Epitomics, #1782-1, 1/100), and visualized using a Cy3-conjugated anti-guinea pig IgG secondary antibody or FITC-conjugated anti-rabbit IgG secondary antibody (Jackson Immunoresearch, 1/200) as appropriate. After the second staining step, the EBs were washed and covered with antifade reagent containing DAPI (Molecular Probe). Images were then captured using a FLUOVIEW FV1000 confocal microscope (Olympus) with 10×, 40× and 100× objectives. All experiments repeated at least two times.

### Measurement of C-peptide, glucagon and somatostatin secretion from EBs

After culturing EBs for 17–18 days in Protocol #3 medium, with or without Dox (1 µg/ml), the medium was changed to fresh IMDM/F12/BSA/B27(−RA) media, with 2 mM glutamine and no growth factors. The EBs were then incubated for 24 hours as indicated, after which the conditioned medium was collected for secretion assays. Concentrations of glucagon and somatostatin in the conditioned medium were measured using enzyme immunoassays (EIAs) specific for glucagon (Yanaihara) or somatostatin (Phoenix Pharmaceuticals) according to the manufacturer's instructions. To assay C-peptide secretion, day 18 EBs were washed and then incubated for 1 hour in Krebs-Ringer bicarbonate HEPES buffer (2 mM glucose) with or without stimulation with secretagogues. C-peptide in the supernatant was then measured using a specific radioimmunoassay (RIA; Linco). The amount of protein in each sample was determined using BCA assays (Pierce), and measured levels of secreted C-peptide, glucagon and somatostatin were normalized to the total protein.

## Supporting Information

Figure S1 **Time course of pancreatic gene expression.** Tet-pdx1/ngn3 ES cells were cultured according to Protocol #2 with BMP4 and cultured in suspension. *Pdx1* and *Ngn3* were induced with or without Dox starting at day 4, and cells were harvested at the indicated time points. Various pancreatic related-genes were analyzed by microarrays. Dox(−); open squares, Dox(+); closed circles.
(TIF)

Table S1 **Protocol for culture conditions.**
(RTF)

Table S2 **Oligonucleotide primers used for PCR**.
(RTF)

Table S3 **Microarray gene expression analysis.** Tet-pdx1/ngn3 ES cells were cultured according to Protocol #2 with BMP4 and cultured in suspension. *Pdx1* and *Ngn3* were induced with or without Dox starting at day 4, and cells were harvested at the indicated time points. RNA samples were isolated from day 13 EBs with or without Dox, βTC6 cells and e15.5 mouse embryo and various pancreatic related-genes were analyzed by microarrays.
(RTF)
