# Supplementary figures and images for: Pdx1 and Ngn3 Overexpression Enhances Pancreatic Differentiation of Mouse ES Cell-Derived Endoderm Population
Source: PLoS One. 2011 Sep 13;6(9):e24058. doi: 10.1371/journal.pone.0024058 (PMC3172220; doi:10.1371/journal.pone.0024058)

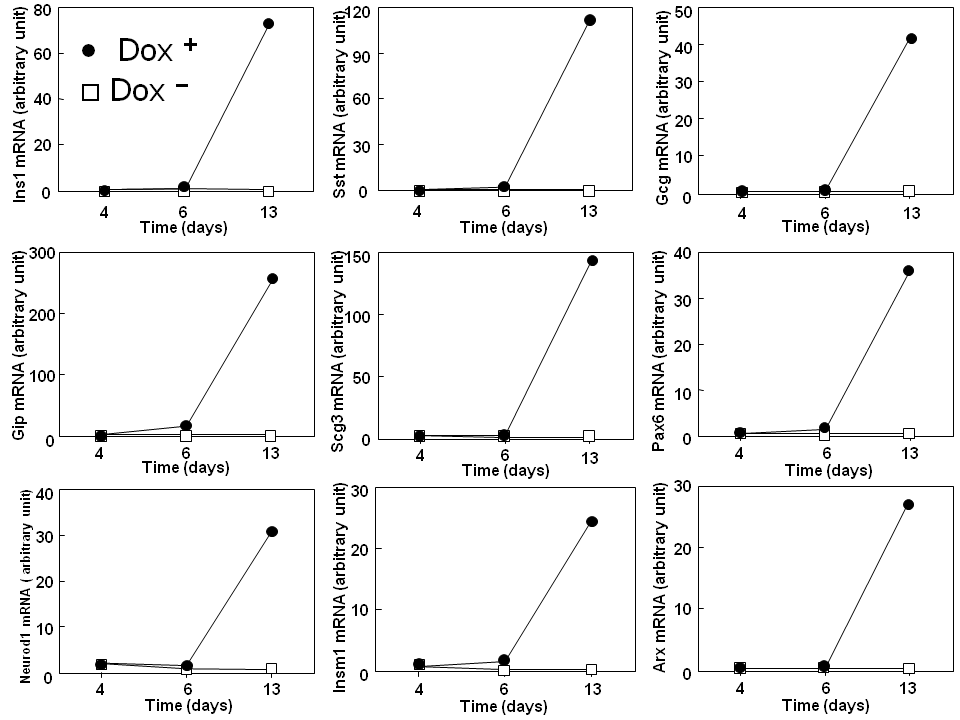

Supplement: Figure S1 — Time course of pancreatic gene expression. Tet-pdx1/ngn3 ES cells were cultured according to Protocol #2 with BMP4 and cultured in suspension. Pdx1 and Ngn3 were induced with or without Dox starting at day 4, and cells were harvested at the indicated time points. Various pancreatic related-genes were analyzed by microarrays. Dox(−); open squares, Dox(+); closed circles. (TIF) [file pone.0024058.s001.tif]
