# Supplementary material for: Pdx1 and Ngn3 Overexpression Enhances Pancreatic Differentiation of Mouse ES Cell-Derived Endoderm Population
Source: PLoS One. 2011 Sep 13;6(9):e24058. doi: 10.1371/journal.pone.0024058 (PMC3172220; doi:10.1371/journal.pone.0024058)
Supplement: Table S1 — Protocol for culture conditions. (RTF) [file pone.0024058.s002.rtf]

�@	Protocol #1 (Figure 1)	Protocol #2 (Figures 2, 3)	Protocol #3 (Figures 4, 5)	Protocol #4 (Figure 6)	
day0	SP34 media	IMDM/F12/BSA/N2,B27(+RA)	IMDM/F12/BSA/B27(-RA)	DMEM/BSA/B27(-RA)	
�@	0.5 mM ascorbic acid 	0.5 mM ascorbic acid 	0.5 mM ascorbic acid 	0.5 mM ascorbic acid 	
�@	4.5 x 10^-4^ M MTG	4.5 x 10^-4^ M MTG	4.5 x 10^-4^ M MTG	4.5 x 10^-4^ M MTG	
�@	�@	�@	�@	�@	
day2	IMDM/SR (15%)	IMDM/F12/BSA/N2,B27(+RA)	IMDM/F12/BSA/B27(-RA)	DMEM/BSA/B27(-RA)	
�@	0.5 mM ascorbic acid 	0.5 mM ascorbic acid 	0.5 mM ascorbic acid 	0.5 mM ascorbic acid 	
�@	4.5 x 10^-4^ M MTG	4.5 x 10^-4^ M MTG	4.5 x 10^-4^ M MTG	4.5 x 10^-4^ M MTG	
�@	100ng/ml Activin A	50ng/ml Activin A	50ng/ml Activin A	50ng/ml Activin A	
�@		�@	�@	�@	
day4		reaggregated	reaggregated	reaggregated	
	�@	IMDM/F12/BSA/N2,B27(+RA)	IMDM/F12/BSA/B27(-RA)	DMEM/BSA/B27(-RA)	
�@	�@	0.5 mM ascorbic acid 	0.5 mM ascorbic acid 	0.5 mM ascorbic acid 	
�@	�@	4.5 x 10^-4^ M MTG	4.5 x 10^-4^ M MTG	4.5 x 10^-4^ M MTG	
�@	�@	50ng/ml Activin A	50ng/ml Activin A	50ng/ml Activin A	
�@	�@	10ng/ml bFGF	10ng/ml bFGF	10ng/ml bFGF	
�@	�@	+/- 50ng/ml BMP4	50ng/ml BMP4	50ng/ml BMP4	
�@	�@	+/- Dox	+/-Dox	+/-Dox	
�@	�@	�@	�@	�@	
day6	IMDM/SR (15%)	IMDM/F12/BSA/N2,B27(+RA)	IMDM/F12/BSA/B27(-RA)	DMEM/BSA	
�@	+/-Dox	monolayer or suspension	suspension	suspension	
�@	�@	+/-Dox	+/-Dox	+/-Dox	
�@	�@	�@	�@	+/-B27(-RA)	
day10	Replate on matrigel	�@	�@	�@	
�@	15%FCS IMDM	�@	�@	�@	
�@	+/-Dox	�@	�@	�@	
�@	�@	�@	�@	�@	
day14-26	Harvest	Harvest day9-16	Harvest day 9-18	Harvest day13-26	
					
Supplements:				
	SP34 media, Gibco, cat.10639011			
	F12, Gibco, cat. 11765054			
	Serum Replacement (SR), Gibco, cat. 10828028			
	N2 supplement, Gibco, cat.17502048			
	B27 supplement, with RA, Gibco, cat. 17504044			
	B27 supplement, without RA, Gibco, cat. 12587010			
	Activin A, R&D Systems, cat. 338-AC-025			
	BMP4, R&D Systems, cat.314-BP-010			
	bFGF, R&D Systems, cat. 233-FB-025			
