# Supplementary material for: Pdx1 and Ngn3 Overexpression Enhances Pancreatic Differentiation of Mouse ES Cell-Derived Endoderm Population
Source: PLoS One. 2011 Sep 13;6(9):e24058. doi: 10.1371/journal.pone.0024058 (PMC3172220; doi:10.1371/journal.pone.0024058)
Supplement: Table S2 — Oligonucleotide primers used for PCR. (RTF) [file pone.0024058.s003.rtf]

Supplemental TableS2. Primer list for pancreas related-genes
	Forward	Reverse	
Ins1	TAGTGACCAGCTATAATCAGAG	ACGCCAAGGTCTGAAGGTCC	
Ins2	CCCTGCTGGCCCTGCTCTT	AGGTCTGAAGGTCACCTGCT	
Gcg	CAGAGGAGAACCCCAGATCA	TCATGACGTTTGGCAATGTT	
Sst	GAGGCAAGGAAGATGCTGTC	AGTTCTTGCAGCCAGCTTTG	
Ppy	GGCCCAACACTCACTAGCTC	CCAGGAAGTCCACCTGTGTT	
Ghrl	GAAGCCACCAGCTAAACTGC	CGGATGTGAGTTCTTGCTCA	
Gip	GCAAGATCCTGAGAGCCAAC	TTGTTGTCGGATCTTGTCCA	
Glp1r	TCAGAGACGGTGCAGAAATG	CAAGGCGGAGAAAGAAAGTG	
amy	CATTGTTGCACCTTGTCACC	TTCTGCTGCTTTCCCTCATT	
Ela	GGAACCATCCTGGCTAACAA	CTCAGTTGGAGGCAATGACA	
Alb1	GCTACGGCACAGTGCTTG	CAGGATTGCAGACAGATAGTC	
Afp	CCTGTGAACTCTGGTATCAG	GCTCACACCAAAGAGTCAAC	
Fabp2	GGAAAGGAGCTGATTGCTGTCC	CTTTGACAAGGCTGGAGACCAG	
Shh	TTAAATGCCTTGGCCATCTC	CCACGGAGTTCTCTGCTTTC	
			
Pcsk1	TTGGCTGAAAGGGAAAGAGA	GCTTCATGTGCTCTGGTTGA	
Pcsk2	CTGTGACGGCTATGCTTCAA	AGCTGCAGATGTCCCAGAGT	
Chga	GAGGAGGAAGAGGAGGCTGT	TGTCCTCCCATTCTCTGGAC	
Glut2	CGGTGGGACTTGTGCTGCTGG	CGCAATGTACTGGAAGCAGA	
Gck	GCCTGTGTATGCAACCATTG	CATTTGTGGGGTGTGGAGTC	
Kir6.2	GGCTCCTAGTGACCTGCACCA	CCACAGCCACACTGCGCTTGCG	
			
Foxa2	TGGTCACTGGGGACAAGGGAA	GCAACAACAGCAATAGAGAAC	
Ptfa1	CACGCTACCCTACGAAAAGC	CCTCTGGGGTCCACACTTTA	
Pax4	AAATGGCGCAGGCAAGAGAA	ATGAGGAGGAAGCCACAGGA	
Pax6	CCATCTTTGCTTGGGAAATCCG	GCTTCATCCGAGTCTTCTCCGTTAG	
NeuroD	CTTGGCCAAGAACTACATCTGG	GGAGTAGGGATGCACCGGGAA	
Isl1	AGATATGGGAGACATGGGCGAT	ACACAGCGGAAACACTCGATG	
Nkx2.2	AACCGTGCCACGCGCTCAAA	AGGGCCTAAGGCCTCCAGTCT	
MafA	ATCATCACTCTGCCCACCAT	AGTCGGATGACCTCCTCCTT	
Pdx1	CCACCCCAGTTTACAAGCTC	TGTAGGCAGTACGGGTCCTC	
Ngn3	CTGCGCATAGCGGACCACAGCTTC	CTTCACAAGAAGTCTGAGAACACCAG	
Hex	AAAAGGAAAGGCGGTCAAGT	CTGCTCACAGGAAGTGTCCA	
			
b-actin	ATGAAGATCCTGACCGAGCG	TACTTGCGCTCAGGAGGAGC	
