# Supplementary material for: Pdx1 and Ngn3 Overexpression Enhances Pancreatic Differentiation of Mouse ES Cell-Derived Endoderm Population
Source: PLoS One. 2011 Sep 13;6(9):e24058. doi: 10.1371/journal.pone.0024058 (PMC3172220; doi:10.1371/journal.pone.0024058)
Supplement: Table S3 — Microarray gene expression analysis. Tet-pdx1/ngn3 ES cells were cultured according to Protocol #2 with BMP4 and cultured in suspension. Pdx1 and Ngn3 were induced with or without Dox starting at day 4, and cells were harvested at the indicated time points. RNA samples were isolated from day 13 EBs with or without Dox, βTC6 cells and e15.5 mouse embryo and various pancreatic related-genes were analyzed by microarrays. (RTF) [file pone.0024058.s004.rtf]

Supplement Table S3
Extracellular genes
Gene symbol		Dox(-)	Dox(+)	Dox+/-
ratio	bTC6	pancreas
(e15.5)	Islet
(e15.5)	
Cart	NM_013732	0.3	33.8	125.3	54.1	6.7	8.3	
Cck	NM_031161	0.7	175.9	238.1	365.8	6.8	0.3	
Chga	NM_007693	1.9	105.4	54.6	238.7	15.2	288.2	
Chgb	NM_007694	0.3	6.2	22.8	35.3	1.6	9.9	
Cpa1	NM_025350	0.5	15.1	32.5	0.3	216.9	262.5	
Cpa2	NM_1024698	0.9	15.3	17.3	10.9	216.5	274.1	
Fgf12	NM_010199	0.3	7.6	23.1	30.2	1.6	11.5	
Gcg	NM_008100	0.4	41.5	93.8	136.7	95.0	310.2	
Gdf6	NM_013526	1.0	28.1	29.1	0.3	2.6	0.3	
Ghrl	NM_021488	4.1	34.9	8.5	0.4	20.1	4.4	
Gip	NM_008119	0.6	251.2	402.7	0.5	3.6	0.4	
ins1 and 2	NM_008386
NM_008387	0.3	73.6	272.5	375.6	227.1	281.3	
Pcsk1	NM_013628	0.5	3.6	7.9	19.8	2.4	45.5	
Pcsk2	NM_008792	2.2	60.9	28.1	195.4	12.9	180.4	
Ppy	NM_008918	1.1	10.3	9.6	62.5	6.4	260.8	
Pyy	NM_145435	1.8	221.9	126.1	6.1	128.1	288.8	
Resp18	NM_009049	0.3	23.4	86.6	234.9	1.3	174.1	
Scg2	NM_009129	0.5	43.7	91.4	241.0	7.7	309.2	
Scg3	NM_009130	0.6	140.4	245.0	249.7	8.9	263.2	
Scg5	NM_009162	0.4	32.1	81.3	74.8	4.4	97.3	
Sct	NM_011328	3.1	116.4	37.6	398.2	1.9	0.3	
Sst	NM_009215	0.3	111.3	412.4	220.0	26.3	288.5	


Nuclear genes
Gene symbol		Dox(-)	Dox(+)	Dox+/-
ratio	bTC6	pancreas
(e15.5)	Islet
(e15.5)	
Arx	NM_007492	0.3	27.6	97.9	0.4	3.3	7.0	
Bhlhb5	NM_021560	0.3	1.6	5.8	0.3	0.3	0.3	
Ebf1	NM_007897	1.2	8.0	6.6	1.1	5.0	1.8	
Ebf3	NM_010096	0.3	9.4	30.4	0.6	2.1	0.3	
Elavl4	NM_1038698	0.3	9.1	33.7	26.6	1.2	7.5	
Hhex	NM_008245	0.6	7.9	13.1	0.3	3.7	1.9	
Id4	NM_031166	0.3	2.7	10.1	0.8	0.9	0.3	
Insm1	NM_016889	0.3	24.6	90.9	73.0	8.2	52.9	
Irx2	NM_010574	0.3	4.0	12.1	1.6	0.9	4.1	
Isl1	NM_021459	1.6	41.3	25.7	120.5	12.4	43.9	
Lmo1	NM_057173	0.7	12.3	18.4	40.4	1.6	1.9	
Myt1	NM_008665	0.4	25.9	65.4	52.5	9.9	23.5	
Neurod1	NM_010894	0.6	30.6	49.4	64.6	4.1	34.8	
Nhlh2	NM_178777	0.3	12.1	44.8	0.7	0.4	0.3	
Nkx2-2	NM_010919	0.3	8.9	32.9	22.1	9.0	13.8	
Nkx6-1	NM_144955	0.3	3.6	11.1	158.9	38.9	193.7	
Pax4	NM_011038	4.1	16.9	4.1	4.2	5.9	2.9	
Pax6	NM_013627	0.3	36.2	127.8	95.2	9.6	62.5	
Pou3f2	NM_008899	0.3	2.6	9.5	0.3	0.3	0.3	
St18	NM_173868	0.3	15.1	55.9	21.1	2.1	23.7	
Tnrc4	NM_172434	0.3	10.9	40.5	5.0	0.7	2.8	
Uncx4.1	NM_013702	0.3	2.6	9.4	0.3	0.3	0.3	


Cytoskeletal & membrane genes
Gene symbol		Dox(-)	Dox(+)	Dox+/-
ratio	bTC6	pancreas
(e15.5)	Islet
(e15.5)	
Astn1	NM_007495	0.3	24.8	92.0	35.9	1.1	0.9	
Chodl	NM_139134	0.3	21.6	68.1	0.6	7.4	0.6	
Dcx	NM_010025	0.3	43.9	162.6	68.5	5.5	4.6	
Dner	NM_152915	0.3	10.9	40.4	6.1	0.4	4.1	
Dpp6	NM_010075	0.4	13.3	38.4	10.2	0.8	2.1	
Drd1ip	NM_026769	0.3	22.8	84.4	19.4	0.6	3.8	
Ecel1	NM_021306	0.3	18.5	68.3	15.3	2.7	0.3	
Gcgr	NM_008101	0.8	15.5	20.3	0.3	1.2	16.7	
Gja1	NM_010288	2.3	2.2	1.0	0.4	0.9	0.3	
Gja7	NM_008122	8.2	10.8	1.3	0.8	5.2	1.2	
Gja9	NM_010290	0.2	3.2	13.7	1.0	0.4	6.3	
Glra2	NM_183427	0.3	9.5	35.3	0.3	0.3	0.3	
IA2	NM_008985	2.2	61.8	28.1	169.1	5.1	109.0	
Lin7a	NM_1033223	0.3	13.3	43.7	7.3	0.8	0.7	
Mast1	NM_019945	0.3	11.4	36.3	1.7	0.4	3.4	
Mmd2	NM_175217	0.3	16.2	50.2	31.1	7.6	0.9	
Pld5	NM_176916	0.3	11.7	34.2	5.2	0.6	0.5	
Rimbp2	XM_132396	0.8	42.1	50.4	155.9	21.3	97.0	
Sez6l2	NM_144926	1.7	58.1	33.8	153.5	11.5	107.0	
Stmn2	NM_025285	0.3	37.9	129.0	9.1	5.6	6.1	
Stmn3	 NM_009133	0.3	38.0	140.5	73.9	1.1	8.1	
Stmn4	NM_019675	0.3	33.1	122.4	8.2	0.5	0.5	
Tmem27	NM_020626	1.6	47.8	29.6	67.8	10.0	119.0	
Tubb3	NM_023279	0.3	11.6	42.9	3.2	0.4	0.7	


Cytoplasmic & signaling genes
Gene symbol		Dox(-)	Dox(+)	Dox+/-
ratio	bTC6	pancreas
(e15.5)	Islet
(e15.5)	
Gng3	NM_010316	0.3	42.2	130.6	10.4	2.2	2.7	
Calb1	NM_009788	0.3	33.7	125.0	6.9	1.1	40.3	
Dcamkl1	NM_019978	0.3	18.0	66.5	14.1	0.9	3.3	
Cryba2	NM_021541	0.3	18.8	58.7	91.8	19.7	30.6	
Celsr3	NM_080437	0.3	14.9	55.3	9.1	2.1	12.9	
Lin7a	NM_001033223	0.3	13.3	43.7	7.3	0.8	0.7	
Grin3a	XM_205495	0.4	16.6	41.7	0.7	3.5	0.3	
Sncg	NM_011430	0.3	9.1	33.5	0.3	1.6	13.8	
Plcxd3	NM_177355	0.3	8.5	31.4	17.0	1.3	7.7	
Gck	NM_010292	2.4	25.4	10.5	10.9	8.5	31.7	
Epha5	NM_007937	0.5	1.3	2.6	2.2	1.2	7.3	
Efna5	NM_010109	2.9	3.0	1.1	2.4	1.9	1.6	
